# Hereditary Hyperferritinemia

**DOI:** 10.3390/ijms24032560

**Published:** 2023-01-29

**Authors:** Alberto Piperno, Sara Pelucchi, Raffaella Mariani

**Affiliations:** 1 Centre for Rare Disease—Disorders of Iron Metabolism, Fondazione IRCCS, San Gerardo dei Tintori, European Reference Network—EuroBloodNet, 20900 Monza, Italy; 2 Centro Ricerca Tettamanti, 20900 Monza, Italy; 3 School of Medicine and Surgery, University of Milano-Bicocca, 20900 Monza, Italy

**Keywords:** ferritin, hyperferritinemia, iron, hereditary

## Abstract

Ferritin is a ubiquitous protein that is present in most tissues as a cytosolic protein. The major and common role of ferritin is to bind Fe^2+^, oxidize it and sequester it in a safe form in the cell, and to release iron according to cellular needs. Ferritin is also present at a considerably low proportion in normal mammalian sera and is relatively iron poor compared to tissues. Serum ferritin might provide a useful and convenient method of assessing the status of iron storage, and its measurement has become a routine laboratory test. However, many additional factors, including inflammation, infection, metabolic abnormalities, and malignancy—all of which may elevate serum ferritin—complicate interpretation of this value. Despite this long history of clinical use, fundamental aspects of the biology of serum ferritin are still unclear. According to the high number of factors involved in regulation of ferritin synthesis, secretion, and uptake, and in its central role in iron metabolism, hyperferritinemia is a relatively common finding in clinical practice and is found in a large spectrum of conditions, both genetic and acquired, associated or not with iron overload. The diagnostic strategy to reveal the cause of hyperferritinemia includes family and personal medical history, biochemical and genetic tests, and evaluation of liver iron by direct or indirect methods. This review is focused on the forms of inherited hyperferritinemia with or without iron overload presenting with normal transferrin saturation, as well as a step-by-step approach to distinguish these forms to the acquired forms, common and rare, of isolated hyperferritinemia.

## 1. Introduction

Many conditions, genetic or acquired, can lead to hyperferritinemia, which is a frequent finding in clinical practice. Increased serum ferritin might be found in patients with or without iron overload and with or without increased transferrin saturation (TSAT) and often requires an extensive diagnostic work-up [1,2,3]. The diagnostic strategy includes collection of family and personal medical history, laboratory and genetic tests, abdominal ultrasound, and eventually evaluation of liver iron content by direct (biopsy) or indirect (quantitative magnetic resonance) methods [1]. Despite this complex and time-consuming approach, the exact etiology often remains unclear. This review is focused on the forms of inherited hyperferritinemia with or without iron overload presenting with normal transferrin saturation (TSAT) (from now on, defined as isolated hyperferritinemia), as well as a step-by-step approach to distinguish these forms from the acquired forms, common and rare, of isolated hyperferritinemia.

## 2. Tissue Ferritin

Ferritin is a ubiquitous protein found in most living organisms, excluding yeasts, and is probably the most abundant and ancient molecule in iron homeostasis. Ferritin is present in most tissues and is mainly located in the cytoplasm, although a mitochondrial form has also been described [4].

### 2.1. Function

The main function of ferritin is to bind Fe^2+^, oxidize it and sequester it in a safe form in the cell, and release iron according to cellular needs. Ferritin is a spherical shell with a central cavity that might contain up to 4500 atoms of iron; it is a 24-subunit heteropolymer consisting of two types of subunits, H (heavy) and L (light), encoded by two different genes located on chromosomes 11q and 19q, respectively [5]. The ratio of H to L subunits within the assembled ferritin protein varies according to tissue type and developmental stage [4,6]. The L subunit, which is more stable and can contain more iron, predominates in tissues with a main storage function (liver and spleen), while the H-subunit, which has significant antioxidant activity, is more common in tissues with high iron oxidation activity (heart and brain). The two subunits are highly conserved throughout evolution, but only H-ferritin has ferroxidase activity. Ferritin stores iron in a mineralized, non-toxic state, but iron bound to ferritin cannot be directly utilized by the cell. To be biologically useful, iron must be released from ferritin, and this mainly occurs through proteolytic degradation of the ferritin shell in the lysosome [7,8]. Ferritin degradation depends on autophagy, a conserved catabolic cellular process that addresses cellular proteins and damaged organelles to be degraded via the lysosome as part of a recycling and protective process that ensures cellular homeostasis. Mancias et al. [9] have identified NCOA4 as a cargo receptor that transmits ferritin to the autophagosome for lysosomal delivery of NCOA4-ferritin complexes, leading to degradation of ferritin in a process known as “ferritinophagy”. NCOA4 levels and ferritinophagy are regulated by intracellular iron levels, and intracellular iron homeostasis is maintained by facilitating storage or release of ferritin as needed. Moreover, NCOA4-mediated ferritinophagy has recently been shown to modulate ferroptosis, an iron-dependent form of regulated cell death characterized by lipid peroxidation [10]. NCOA4 deletion inhibits ferroptosis by blocking ferritinophagy and ferritin degradation, while NCOA4 overexpression increases sensitivity to ferroptosis. Since ferroptosis is associated with various pathological conditions and NCOA4 and ferritinophagy can modulate ferroptosis, there is interest in understanding their relative role in pathogenesis of diseases, such as neurodegeneration, cancer, and infections [8].

### 2.2. Synthesis

Ferritin synthesis is controlled at both transcriptional and post-translational levels. Iron tightly regulates ferritin synthesis at the post-translational level through a well characterized mechanism of coordinate cytosolic regulation of gene expression that is described in detail in previous reviews [11,12]. Briefly, H- and L-ferritin mRNAs contain a specific structure in the 5′UTR named the iron-responsive element (IRE) that binds with high-affinity iron regulatory proteins (IRP1 and IRP2). IRE–IRP interactions regulate expression of other mRNA-encoding proteins involved in iron acquisition (transferrin receptor 1, divalent metal transporter (1), utilization (erythroid 5′-aminolevulinic acid synthase, mitochondrial aconitase, Drosophila succinate dehydrogenase, hypoxia-inducible factor (2), and export (ferroportin) [11]. Additional mRNAs are regulated by IRPs, including α-synuclein and amyloid precursor protein [13]. The IRE binding activity of IRPs is modulated by iron (IRP1) and the redox status (IRP2) of the cell. Under conditions of high iron availability, IRP1 forms an iron–sulfur cluster and acquires aconitase activity while losing RNA-binding activity and increasing ferritin synthesis, while the opposite occurs under low iron availability [11,12]. In contrast, IRP2 does not contain an iron–sulfur cluster and is regulated by ubiquitination and proteasomal degradation by an E3 ubiquitin ligase complex that contains an F-box protein, FBXL5 [14]. In addition to iron, ferritin synthesis is regulated by heme at both transcriptional (via Bach1 binding) and translational levels (via IRP2 binding) [15], and cytokines during development, cellular differentiation, proliferation, and inflammation at transcriptional, post-transcriptional, and translational levels. Cytokine-dependent control is particularly relevant to inflammation because ferritin is an acute phase reactant [16]. Ferritin synthesis is induced by IL-1β, IL-6, or TNFα in the HepG2 hepatic cell line, and iron is required for this regulation [16,17]. Lipopolysaccharide (LPS; endotoxin), a component of the outer membrane of Gram-negative bacteria, also induces ferritin synthesis. Moreover, inflammation increases ferritin synthesis indirectly by inducing hepcidin transcription through the IL-6–IL-6 receptor–STAT3 pathway, thus limiting intestinal iron absorption and iron release from macrophages [16,18]. In addition, activation of cytokines down-regulates ferroportin synthesis. All in all, they induce accumulation of iron in macrophages and stimulate ferritin synthesis via the classical IRE–IRP regulatory system [16]. Extreme manifestations of ferritin as an acute phase reactant are four uncommon conditions that are associated with very high ferritin levels: macrophage activation syndrome (MAS), adult onset Still’s disease (AOSD), catastrophic antiphospholipid syndrome (cAPS), and septic shock [17]. It has been suggested that they be grouped together under the term “hyperferritinemic syndrome” because of the hypothesis that the very high ferritin levels are not just the product of inflammation but may play a pathogenic role [17].

Hormonal pathways are also involved in ferritin regulation. Thyroid hormone may regulate ferritin post-transcriptionally: T3 modulates activity of IRP1, affecting its ability to bind to ferritin IRE, possibly inducing phosphorylation of IRP1; T3 and TRH also induce phosphorylation of IRP2, resulting in ferritin synthesis increase. In addition to thyroid hormone, insulin and IGF-1 have also been implicated in regulation of ferritin at the mRNA level [19]. In human hepatoblastoma cell line HepG2, alcohol was able to induce, in a specific manner, ferritin expression [20]. A distinct association between alcohol intake and serum ferritin levels was demonstrated in a population survey including 2235 healthy Danish individuals [21].

## 3. Serum Ferritin

**Figure 1:**
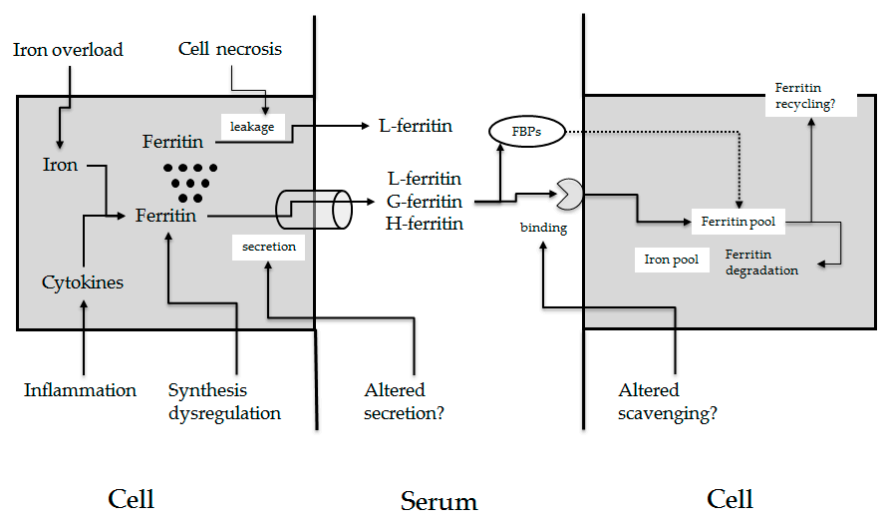
Physiopathology of hyperferritinemia. Iron modulates ferritin at post-transcriptional level, cytokines at transcriptional, post-transcriptional, and transductional level, and by inducing macrophage iron retention through hepcidin up-regulation. Other factors that can induce ferritin synthesis (alcohol, thyroid hormones) are not reported in the figure (see text). All these factors cause increased secretion of ferritin and hyperferritinemia. Mutations in the IRE region of *FTL* cause dysregulated constitutive synthesis of L-ferritin and hyperferritinemia, while mutations in the first exon of *FTL* cause hyperglycosylation of ferritin and altered/increased secretion or reduced ferritin clearance. Hepatocellular necrosis occurring in acute and chronic liver diseases of any cause caused increased tissue ferritin leakage by the liver. Circulating ferritin is cleared by binding to specific receptors or ferritin-binding proteins (FBP). Inside the cell, ferritin can be degraded or possibly recycled in the blood by specialized cells involved in ferritin secretion process.

Since 1972, it was demonstrated that ferritin could be reliably detected in human serum and that it was elevated in patients with iron overload and decreased in patients with iron deficiency diseases [22]. In 1975, Jacobs and Worwood [23] suggested that a serum assay might provide a “useful and convenient method of assessing the status of iron storage”. Accordingly, serum ferritin measurement has become a routine laboratory test, although it is now known that many additional factors, including inflammation, infection, metabolic abnormalities, and malignancy—all of which may elevate serum ferritin—complicate interpretation of this value. In addition, several inherited defects of the L-ferritin gene (*FTL*) can lead to either inappropriately high or low ferritin levels [24]. Figure 1 summarizes the various factors that may influence serum ferritin level; however, despite this long history of clinical use, fundamental aspects of the biology of serum ferritin are still unclear.

### 3.1. Characteristics

Ferritin is present at a considerably low proportion in normal mammalian sera compared to tissues (less than 0.1% of the estimated total body ferritin) [6]. Serum ferritin is relatively low in iron [25] and in humans seems to consist of L-ferritin, a glycosylated form of L called G-ferritin, and traces of H-ferritin [26]. In mice, serum ferritin consists mainly of L-subunits, contains few H-subunits, and small amounts of iron as in humans, whereas glycosylated ferritin is undetectable and L-subunits are often truncated at the C-terminus (S-ferritin), resulting in a characteristic 17-kD band previously observed in lysosomal ferritin [27]. Due to the low iron content, serum ferritin is not thought to be an important source of hepatic iron in either healthy people or patients with iron-overload diseases. However, Nielsen et al. found a close correlation between serum concentrations of ferritin protein and ferritin iron in patients with varying degrees of iron overload and estimate the proportion of serum ferritin iron to be about 5% of the total plasma iron in patients with moderate liver iron overload [28]. Although this proportion is small, it is not negligible in patients with hyperferritinemia, and the authors speculated that this iron proportion may play a role in recirculation of iron [28].

### 3.2. Source and Secretion

The source and exact pathway of serum ferritin secretion are not yet fully understood. Hepatocytes, lymphoid cells, and, especially, macrophages and Kupffer cells, secrete ferritin [27]. Genetic evidence for the contribution of macrophages to serum ferritin levels comes from cell-type-specific ablations of IRP2, which decrease translational repression of ferritin, leading to overexpression of ferritin. Mice with a macrophage-specific deletion of the IRP2 gene have elevated serum ferritin levels, whereas neither mice with intestinal epithelial nor a hepatocyte-specific ablation of IRP2 have elevated serum ferritin levels [29]. Although serum ferritin is generally thought to reflect body iron status, these results support the notion that serum ferritin more specifically reflects macrophage iron status. Mammalian ferritins do not have signal peptides for the classical endoplasmic reticulum/Golgi secretion pathway. Instead, a 13 amino-acid motif unique to ferritins has been identified in the BC-loop of H- and L-subunits, which appears to be essential for ferritin secretion [30]. There is evidence that serum ferritin is secreted via a non-classical lysosomal secretion pathway, specifically secretory lysosomes [27]. Recent data suggest that ferritin may be secreted via the endo-lysosomal system, not only via the non-classical secretory-autophagy pathway but also through the multivesicular body exosome pathway [30]. This was supported by results showing that specific defects in early or later stages of vesicle transport to secretory lysosomes or secretory autophagy (LROs) have opposite effects on ferritin secretion.

Ferritin secretion into the medium of cultured cells is increased by iron and cytokines interleukin-1-β (IL-1) and tumor necrosis factor-α (TNF-α) [31], but it is unclear whether this is the result of increased synthesis of ferritin in the cell or if they also up-regulate ferritin secretion. In normal conditions, secreted ferritin contributes substantially to the serum ferritin pool, whereas ferritin leakage from cells contributes only slightly to serum ferritin concentration under normal conditions. However, in patients with acute or chronic liver diseases, there is increased release of tissue ferritin from damaged hepatocytes into the bloodstream, leading to high or very high serum ferritin concentrations [32].

### 3.3. Function

Although the biological significance of serum ferritin is still not clear, recent studies have provided evidence that extracellular ferritin can act as an iron carrier to supply cells with iron through interaction with specific receptors. There is experimental evidence that iron in the form of ferritin can be released by macrophages and taken up by cell types that are specialized in storing iron, such as hepatocytes, or that consume large amounts of iron, such as developing erythroid cells [33,34]. Indeed, previous studies have shown that ferritin can bind to a variety of different cell types. Cells take up ferritin through two pathways: direct uptake by ferritin receptors and indirect uptake by ferritin-binding proteins (FBPs) [35,36,37]. Fargion et al. identified a saturable binding site specific for H ferritin on the surface of human lymphocytes [38]. Specific and saturable binding of H ferritin has also been observed in liver cells, brain oligodendrocytes, enterocytes, and erythroid progenitors cells [39]. The presence of ferritin receptors, such as murine TIM2, human TfR1, and SCARA5, on these cells [39,40,41] supports the hypothesis that ferritin is an iron donor protein. TIM-2 is a transmembrane protein expressed in mouse liver, kidney, T cells, and B cells [40,42]. It has no known human orthologue and specifically binds ferritin H but not ferritin L. SCARA5 is a scavenger receptor that mediates uptake of ferritin iron (with a preference for L-chain enriched ferritin), which is required for specific cell types in the developing kidney [41]. SCARA5 has relatively wide tissue distribution and could internalize ferritin for either ferritin removal or iron delivery. It may be involved in regulation of ferritin homeostasis and cell death, but further studies are needed to understand the exact role of the receptor in these pathways [43]. More recently, Li et al. [39] identified human TfR1 as another cell surface receptor for H ferritin, while no binding to L ferritin was observed.

Anti-ferritin autoantibodies, high-molecular-weight kininogen (HK), and fibrinogen are probably the most common human FBPs [37]. One of the suggested physiological roles of FBPs is to remove ferritin from the bloodstream to protect against oxidative stress [37]. In addition, binding of FBPs to ferritin can affect blood coagulation and influence iron metabolism, oxidative state, angiogenesis, inflammatory condition, and immune response. HK is a protein that interacts with ferritin and plays a role in blood clotting, blood pressure regulation, and inflammation. HK is cleaved by serine protease kallikrein to form two independently active proteins: bradykinin (BK) and two-chain high-molecular-weight kininogen (HKa). Ferritin interacts directly with both HK and even more efficiently with HKa. As a result, ferritin abolishes the anti-angiogenic effect of HKa, leading to increased blood vessel growth [44]. The pro-angiogenic activity of ferritin exerted by its ability to bind HK/HKa may represent a physiological response in the context of inflammation and wound healing but also a pathological response in the context of tumor growth.

Other functions have been proposed for serum ferritin concerning its role as a signaling molecule and immunity [6]. Ruddell et al. [45] proposed a novel role for extracellular ferritin as a pro-inflammatory signaling molecule in hepatic stellate cells via an iron-independent pathway. Previous in vitro studies suggested that ferritin may modulate immune response by inhibiting lymphocyte function [46]. Later, Broxmeyer et al. found that H- ferritin, but not the L subunit, inhibited proliferation of granulocyte, macrophages, erythrocytes, and multipotent progenitor cells [47]. Li et al. [48] showed that ferritin H plays an important role in chemokine-receptor-mediated signal transduction and migration, effects that may contribute to the immunomodulatory activity of ferritin. Very recently, it was shown that H-ferritin may modulate macrophage response to immune stimuli, playing a relevant role in protection against iron-induced oxidative stress and cell death [49]. Other potential functions of serum ferritin as a modulator of immunity and autoimmunity are described elsewhere [16,50,51].

### 3.4. Clearance

In rats, dogs, and rabbits, ferritin is cleared from the bloodstream very rapidly (T½ 2–10 mm for doses of about 1 μg), whereas, in humans, the overall clearance rate of labelled ferritin was relatively slow, with 50% of the original radioactivity remaining in the plasma after 30 h [52]. The authors suggest that glycosylation is an important factor prolonging the survival of ferritin, possibly explaining the very different excretion rates in experimental animals and humans. There is little information on the fate of serum ferritin after clearance. It could be subject to degradation, but it cannot be ruled out that it may be recycled via the non-classical lysosomal secretion pathway.

## 4. Diagnosis of Hyperferritinemia

Because of the multitude of factors involved in regulation of ferritin synthesis, secretion, and uptake, and because of its central role in iron metabolism, it is not surprising that hyperferritinemia is a relatively common finding in clinical practice, occurring in a wide range of conditions both genetic and acquired, and with or without iron overload (Table 1). Epidemiological studies conducted in North American Caucasians estimated the prevalence of hyperferritinemia (ferritin > 300 μg/L in men and >200 μg/L in women) to be 19.6% in men [53]. In women, a large age-dependent distribution of ferritin due to menstrual losses and pregnancies was found, ranging from 3% in pre-menopausal women to 17% in those over 70 years of age [53]. These figures show that hyperferritinemia can have a significant impact on the health system both at the general practitioner level and in secondary (hematology units) or tertiary centers (centers for iron metabolism disorders). Therefore, a careful strategy is required in differential diagnosis of hyperferritinemia that includes considering family and personal history and evaluating various biochemical and instrumental tests [1,2,3]. A basic approach to hyperferritinemia is evaluation of transferrin saturation. A transferrin saturation (TSAT) greater than 45% is generally considered an appropriate threshold for suspecting iron overload caused by increased intestinal iron absorption [1,54,55,56] and is the first and indispensable test in differential diagnosis of hyperferritinemia. Table 1 summarizes the various causes and pathogenesis of hyperferritinemia subdivided according to the presence of normal or elevated TSAT.

Flowcharts have been proposed in previous reviews and guidelines to simplify the diagnostic pathway in patients with hyperferritinemia with and without an elevated TSAT [1,2,3,54,55]. The presence of an elevated TSAT generally indicates primary- or secondary-induced increased intestinal iron absorption and iron release from macrophages, leading to occurrence of non-transferrin-bound iron (NTBI) and labile plasma iron (LIP), cellular iron accumulation and iron-induced ferritin synthesis, and hyperferritinemia [57]. In most cases, the pathophysiological mechanism is inhibition of hepcidin synthesis caused by altered regulation (hemochromatosis) [58], ineffective erythropoiesis (iron loading anemias) [59], or protein synthesis deficiency, as observed in advanced liver disease of any cause [60]. Decreased synthesis of transferrin in these cases may also contribute to development of NTBI and LIP, while transfusions in early stages lead to macrophage iron overload and increased serum ferritin with normal TSAT; with increasing transfusion load, iron accumulation overwhelms the capacity of the macrophages, leading to increased TSAT and, thus, parenchymal iron overload [61]. For a detailed description of the forms of hyperferritinemia associated with increased TSAT, we refer the readers to other reviews [54,55].

## 5. Isolated Hyperferritinemia

In a subanalysis of data from the Hemochromatosis and Iron Overload Screening Study (HEIRS), it was shown that only about 13% of subjects with hyperferritinemia had an elevated TSAT, suggesting that isolated hyperferritinemia accounts for almost 90% of all cases of hyperferritinemia [53]. Because of the high prevalence, many patients are now referred to specialists for isolated hyperferritinemia, which often causes concern among patients and their general practitioners. Patients are often told that they may have hemochromatosis and iron overload. Some surf the internet and are alarmed at the alleged link between hyperferritinemia and neoplasia. Others are mistakenly induced to have a phlebotomy, with the risk of developing iron deficiency anemia, or to HFE genetic testing, with the risk of identifying HFE genotypes at no- or low-risk and misdiagnosing hemochromatosis. Finally, some repeat ferritin testing every six months or annually without reaching any diagnostic conclusions.

**Figure 2:**
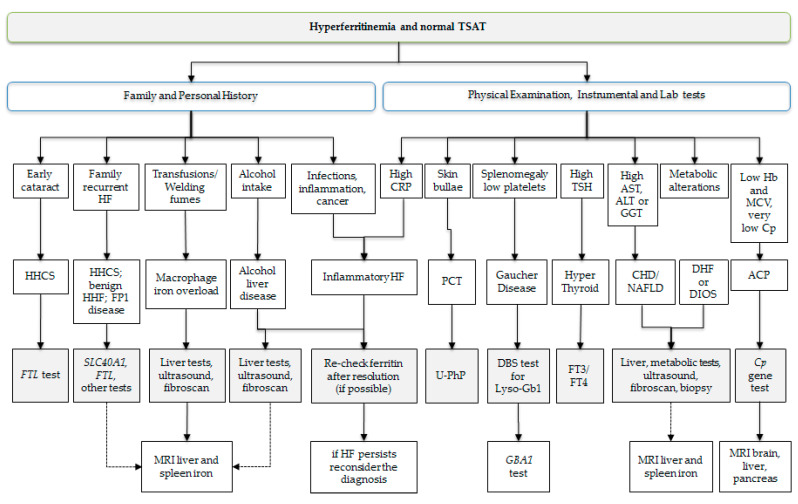
Flow-chart of isolated hyperferritinemia. In the first step, information on patient and family history, clinical symptoms, and laboratory tests can be used to hypothesize or exclude some causes of hyperferritinemia and propose other laboratory or instrumental tests to further proceed in the step-by-step differential diagnosis. The second step shows the hypothetical diagnosis that should be confirmed or supported by more specific biochemical, genetic, and instrumental tests. Genetic tests can be performed to confirm the suspect of HHCS in case of a personal and family history of early cataract or hyperferritinemia, aceruloplasminemia in case of absent or very low level of serum ceruloplasmin, Gaucher disease in case of positive DBA test for LysoGb1, and ferroportin disease in case of recurrent hyperferritinemia with dominant transmission. Liver ultrasound and fibroelastometry (Fibroscan^®^) are often part of the diagnostic evaluation to assess the presence of steatosis and fibrosis/cirrhosis. Quantification of liver and spleen iron by MRI can be requested for assessment of iron status in patients with history of repeated transfusion, prolonged exposure to welding fumes, or in chronic liver diseases, including dysmetabolic iron overload, ferroportin disease, and aceruloplasminemia.

Figure 2 shows a simple flow-chart based on a clinical–laboratory approach used in the tertiary center of S. Gerardo Hospital in Monza for diagnosis of isolated hyperferritinemia. As a first step, personal and family history, physical examination, and available laboratory and instrumental tests must be carefully evaluated to ascertain useful elements for guiding the diagnosis.

### 5.1. Family and Personal History

It is important to note a recurrent family history of hyperferritinemia as some of the known hereditary forms of isolated hyperferritinemia have a dominant phenotype. These include hereditary hyperferritinemia cataract syndrome (HHCS) due to mutations in the IRE region of *FTL*, so-called benign hyperferritinemia due to mutations in the first exon of *FTL*, and ferroportin disease due to loss-of-function mutations of *SLC40A1* [24,62,63]. Although there are few reports of de-novo mutations [64,65], a positive family history of hyperferritinemia is often found in these cases. A personal history of at least ten blood transfusions may explain the presence of hyperferritinemia and iron overload. This is often the case in patients who have undergone multiple transfusions due to hematopoietic stem-cell transplantation or patients who have been in emergency departments for a long time due to severe sepsis, polytrauma, or other causes [66]. In these patients, iron overload varies according to number of transfusions performed, leading initially to a predominant accumulation of iron in the macrophage, which spreads to the hepatocyte and portal vein area when the macrophages become saturated [61,66]. A history of prolonged exposure to welding fumes should also be considered as a recent report shows that a subset of welders with prolonged exposure to welding fumes, and especially those using fewer protective devices, develop severe systemic iron overload [67]. A history of active inflammatory disease or cancer, recent infection, trauma, or strenuous physical activity may be causes of hyperferritinemia, which should be checked at a distance from the resolution of the problem. Although hyperferritinemia can be found in patients with advanced neoplasia, it cannot be used as a predictive index of cancer risk [68,69]. Therefore, once the necessary tests for screening hyperferritinemia have been performed, it is not necessary to conduct further investigations in search of an occult neoplasm unless some of the tests performed suggest it. High alcohol intake can be an independent cause of hyperferritinemia, although this habit is often associated with other inadequate dietary lifestyles contributing to hyperferritinemia [20,21]. Unless there are clinical conditions requiring immediate intervention, it is good practice to reassess iron indices after at least six months of lifestyle change.

### 5.2. Physical Examination, Instrumental, and Laboratory Tests

Given the strong association between metabolic changes and hyperferritinemia, which account for a large proportion of isolated hyperferritinemias [70,71], measuring weight and height and calculating BMI and/or measuring abdominal circumference are crucial aspects of assessment of a patient with hyperferritinemia. Other useful indicators that may result from careful physical examination are those suggestive of chronic liver disease, such as presence of hepatosplenomegaly, and indirect signs of advanced liver disease (palmar erythema, spider nevi), and those suggestive of porphyria cutanea tarda (PCT), such as vesicles and blisters or their appearance in areas exposed to sunlight [72]. In patients with hyperferritinemia, some general tests are usually already performed, which may be useful in differential diagnosis:

- blood count, which may indicate aceruloplasminemia in the case of mild microcytic anemia [73] or Gaucher’s disease in the case of thrombocytopenia (even more so in the presence of concomitant splenomegaly) [74];
- metabolic indices (glycemia, HDL cholesterol, triglycerides), which, together with overweight/obesity and arterial hypertension, may indicate forms of dysmetabolic hyperferritinemia (DHF) or iron overload (DIOS) [70];
- liver tests (AST, ALT, γGT) to suspect chronic liver disease of any cause;
- inflammatory indices (CRP and protein electrophoresis) to support cytokine-induced hyperferritinemia [75];
- abdominal ultrasound to determine the presence and extent of hepatic steatosis, which, together with liver and metabolic tests, may lead to diagnosis of non-alcoholic fatty liver disease (NAFLD), commonly associated with hyperferritinemia [76], or indirect signs of liver cirrhosis [77].

If these tests are not performed, they must be requested or repeated with addition of the ceruloplasmin dosage. Other tests may be requested based on available clinical and laboratory data: porphyrin urine if PCT is suspected, basal and post-prandial insulinemia or oral glucose-tolerance test (OGTT) with measurement of basal and two-hours insulinemia to identify glucose intolerance, latent diabetes, or hyperinsulinemia, thyroid function tests for possible hyperthyroidism, α-antrypsin, and other liver tests (viral markers, autoimmunity, plasma, and urine copper) for hypertransaminasemia [78], and liver fibroelastometry for suspected advanced liver disease [77,79].

Apart from the rare and more easily identified forms in relation to personal history (multiple transfusions, prolonged exposure to welding fumes), some symptoms (early cataract, skin blisters, and bullae, or those suggestive of hyperthyroidism) and specific tests (ceruloplasmin or porphyrin in the urine, thyroid tests) point to diagnosis; definition of the causes of hyperferritinemia is, in most cases, the result of a complex evaluation often based on exclusion criteria. In this diagnostic process, it may be necessary to make use of further investigations, such as magnetic resonance imaging (MRI) of the liver, spleen, or brain, specific biochemical tests if Gaucher’s disease is suspected, and appropriate genetic tests for diagnostic confirmation of HHCS, other inherited forms of hyperferritinemia, ferroportin disease, aceruloplasminemia, and Gaucher’s disease. Nevertheless, several hyperferritinemias remain unexplained, suggesting that other genetic or non-genetic causes have not yet been identified.

## 6. Inherited Isolated Hyperferritinemias

This group includes disorders with or without iron overload caused by defects in genes involved in iron metabolism (*FTL*, *SLC40A1, CP*), as well as non-iron-related genetic disorders often associated with hyperferritinemia with normal TSAT. Table 2 summarizes the epidemiological and genetic data, as well as the main clinical and biochemical features and therapeutic approach.

### 6.1. Hereditary Hyperferritinemia Cataract Syndrome (HHCS) (OMIM#600886)

HHCS is a rare genetic disease, with widespread worldwide prevalence not yet established [62]. HHCS is characterized by a persistent elevation of serum ferritin regardless of amount of iron stores. Thus, serum iron, TSAT, and body iron are normal. The disease has a dominant phenotype and is caused by mutations (point mutations or deletions) of the IRE CAGUGX hexaloop and stem of *FTL*. The mutations variably disrupt negative control of L-ferritin synthesis at the translational level through IRE–IRP interaction [24], leading to constitutive up-regulation of L-ferritin, intracellular accumulation of ferritin, mainly in the form of the homopolymers H0-L24, and hyperferritinemia [62,80]. There is a close relationship between cellular ferritin and serum ferritin, and the presence of glycosylated subunits is strong evidence that the circulating protein is a by-product of intracellular ferritin synthesis and secretion [81]. Apart from hyperferritinemia, the only consistent abnormality in affected patients is a nuclear cataract, which appears in infancy or before the age of 50 years [80]. Lens opacity probably results from overproduction of L-type ferritin, as shown by the presence of light-diffracting crystalline deposits rich in L-ferritin in cataractous lenses from individuals with HHCS [82]. Ophthalmologic follow-up studies show that lens opacities progress with age. Age of cataract onset and range of ferritin levels vary even in people with the same mutation in the same or different families, confirming the heterogeneity of the phenotype [80]. Although there are no other clinical manifestations associated with the disease, its recognition is important for two reasons: *i.* to enable early detection of cataracts in asymptomatic people, which can be controlled and removed even in childhood; *ii.* to avoid unnecessary diagnostic procedures and sometimes even inadequate treatments. In patients with HHCS, serum ferritin is no longer a reliable indicator of iron stores in both iron deficiency and iron overload, which is something to keep in mind.

### 6.2. Benign Hyperferritinemia (OMIM#600886)

Benign hyperferritinemia is another rare dominant form of inherited hyperferritinemia not associated with iron overload or cataracts caused by mutations in *FTL*. Three mutations in the N-terminal region of *FTL* are known to cause amino acid substitutions at positions 26, 27, and 30 in the heterozygous state in the A helix of L-ferritin, resulting in benign hyperferritinemia [24,83,84]. This ferritin is susceptible to glycosylation, and the degree of glycosylation of serum ferritin is always greater than 90%. The reason for hyperferritinemia and hyperglycosylation associated with mutant ferritin is still unknown. The authors hypothesized that mutations alter the hydrophobicity of ferritin, increasing its glycosylation and cellular secretion into the blood, but delayed clearance may also be a contributing factor [84]. Despite the high degree of glycosylation of serum ferritin, no other side effects have been identified in patients with this disease.

### 6.3. Ferroportin Disease (OMIM #606069)

Ferroportin (FPN1) disease is a rare dominant disease caused by mutations of *SLC40A1*, which encodes for FPN1, the only known mammalian exporter of iron from cells into the blood. Copper ferroxidases (hephestin in enterocytes and ceruloplasmin in macrophages and hepatocytes) are required by FPN1 to release iron to plasma transferrin. Deficiency in these ferroxidases causes cellular iron retention in specific cell types, as shown in hephestin-deficient *sla* mice and in humans with aceruloplasminemia [85]. FPN1 is expressed mainly on the basolateral membrane of hepatocytes, macrophages, and duodenal enterocytes [86]. Hepcidin, the key regulator of iron homeostasis, controls iron levels in the body by binding to FPN1 and blocking it by degradation [87] or via occlusion [88], having a general inhibitory effect on iron release in the body. Excess iron increases hepcidin levels, resulting in decreased expression of FPN1 on cell membranes and limiting iron release into plasma [88]. Mutations in *SLC40A1* can lead to two types of autosomal dominant iron overload disorders according to their loss-of-function or gain-of-function effect. Gain-of-function mutations cause Hemochromatosis type 4 (previously classified as Hemochromatosis type 4B), characterized by resistance to hepcidin and a typical hemochromatosis phenotype (high TSAT and serum ferritin, and parenchymal iron overload) [63]. Loss-of-function mutations are more common and cause FPN1 disease (formerly classified as Hemochromatosis type 4A), which is characterized by hyperferritinemia with normal transferrin saturation (TSAT) and predominant iron accumulation in macrophages [54], leading to increased production of ferritin and its release into plasma. Serum ferritin is often elevated out of proportion to iron stores, supporting the major role of macrophages in secretion of ferritin in plasma. Iron accumulation in hepatocytes and increased TSAT may occur in the late stages of the disease, leading to a mixed form of sinusoidal and hepatocellular iron overload in some patients [63]. FPN1 disease is characterized by phenotype heterogeneity due to reduced stability of some mutants and modifiers that might influence disease severity (gender, age, alcohol abuse, obesity, and metabolic syndrome) [89]. Contrary to aceruloplasminemia, patients with FPN1 disease do not develop iron deficiency anemia. This suggests that, despite the inefficient recycling of iron from the macrophages, the system is still able to compensate for iron demand from bone marrow in a steady state. Only when phlebotomized with the same frequency as in HFE-related hemochromatosis to eliminate iron overload may these patients manifest their inefficient iron absorption and recycling capacity, developing functional iron deficiency anemia [63]. Patients with FPN1 disease usually do not develop significant iron-related manifestations, probably due to predominant iron accumulation in macrophages, which are less susceptible to toxic iron effects than parenchymal cells [63]. However, the disease can often be confused with other causes of hyperferritinemia. Abdominal MRI has been suggested as a useful non-invasive tool for diagnosing the disease as it shows iron retention in both the liver and spleen differently from hemochromatosis and iron loading anemias [90,91]. However, this finding is not specific as it is frequently observed in dysmetabolic hyperferritinemia and dysmetabolic iron overload syndrome [70]. A number of mutations across the *SLC40A1* gene have been identified worldwide, with p.Val162del being the most frequent mutation reported in different populations [63]. Variants in *SLC40A1* are very common in African populations, suggesting that *SLC40A1* is the gene most frequently associated with hereditary hyperferritinemia in Africans [63]. A major pitfall in diagnosis of FPN1 disease is the heterogeneous and unspecific phenotypic expression. Although some studies suggest that FPN1 disease might be more frequent than believed [92], we have shown that only a minority (3.6%) of the patients with isolated hyperferritinemia and iron overload carried a mutation in *SLC40A1* [93]. This figure improves to 8% and 18.2% when only patients with relatives with significant hyperferritinemia are considered, suggesting that an accurate collection of family data increases the power of genetic testing [93]. More recently, in 1306 non-hemochromatosis patients with hyperferritinemia and liver iron overload, only 71 patients (5.4%) showed *SLC40A1* variants, of which 10 were of unknown significance and 22 were likely pathogenic [94]. A multivariate analysis aimed at developing a model in which the possibility of FPN1 disease could be assessed by genetic test results showed that being female, younger, having high ferritin, high liver iron concentration (LIC), and no arterial hypertension or diabetes were significantly associated with diagnosis of the disease. The score based on these five variables showed a negative predictive value of 99.0 (96.9–99.7)% and a positive predictive value of 12.6 (9.4–16.6)%. Therefore, it seems to be useful primarily as a screening tool to rule out the disease and enable more appropriate genetic testing [94]. Indeed, due to heterogeneous expression, FPN1 disease can be overlooked or misdiagnosed, especially with respect to metabolic syndrome. Phlebotomy is also the basis of treatment in FPN1 disease but may be poorly tolerated due to defective iron release from macrophages. Thus, the frequency and targets are quite different from those in hemochromatosis. Although no studies have yet evaluated the optimal schedule, phlebotomy once/twice a month with careful monitoring of serum ferritin and TSAT is recommended to avoid development of iron-restricted erythropoiesis and anemia. A reasonable target for therapy is a serum ferritin level of 100–200 μg/L, to be maintained with a 4–12 month phlebotomy schedule [63].

### 6.4. Aceruloplasminemia (OMIM #604290)

Aceruloplasminemia (ACP) is an ultra-rare recessive genetic disorder caused by defective production of ceruloplasmin (Cp). Cp, along with hephestin and zyklopen, belongs to the multicopper ferroxidase family, which binds FPN to promote cellular iron efflux by oxidizing ferrous iron to the ferric state [95]. Cp exists in a soluble form secreted by the liver but also as a glycosylphosphatidylinositol (GPI)-linked protein in astrocytes and leptomeningeal cells, and also in macrophages, hepatocytes, and in many other tissues [96]. Studies in Cp-KO mice showed impaired hepatocyte and reticuloendothelial iron efflux [97] and demonstrated that Cp-GPI is important for regulating astrocytes iron efflux [98]. In cells expressing Cp-GPI, ferroxidase activity is required to stabilize cell surface FPN1 [99], suggesting that Cp is a second determinant of FPN1 stability after hepcidin. Thus, the absence of Cp-ferroxidase activity in ACP promotes internalization and degradation of FPN1, which favors intracellular iron accumulation. These results suggest that ACP results from an iron imbalance caused by impaired iron efflux rates from storage sites, supporting the notion that tissue damage may depend on two sequential or overlapping events: an iron deficiency condition due to impaired mobilization of iron from macrophages or glial cells to neighboring cells and excessive iron retention and accumulation in parenchymal and neuronal cells, resulting in oxidative damage [98,100]. In addition, Kaneko et al. [101] have shown that hepcidin levels are reduced in ACP, possibly due to iron-restricted erythropoiesis, a factor that may contribute to development of systemic iron overload. For more details on pathogenesis of ACP and Cp function, we refer the readers to previous exhaustive reviews [73,102,103]. Since Cp is involved in iron homeostasis at the systemic and brain levels, ACP is characterized by iron-restricted erythropoiesis on one hand and brain and systemic iron overload on the other. Thus, the classic clinical picture of ACP includes mild microcytic anemia with low serum iron and TSAT levels, hyperferritinemia, diabetes, and retinal and neurological degeneration. Neurologic symptoms usually appear in the fifth decade of life and are wide-ranging, including cerebellar ataxia, involuntary movements, parkinsonism, mood and behavior disorders, and cognitive impairment [104,105]. With increasing use of magnetic resonance imaging (MRI) for neurological diagnosis, a small proportion of patients with neuropsychiatric symptoms have been found to have cerebral iron accumulation, enabling diagnosis of ACP. Although less common, patients with ACP without neurologic symptoms and brain iron overload or with brain iron overload without or with very mild neurologic symptoms have also been reported after the age of 50 years [106,107,108]. This suggests that the phenotype of ACP is more heterogeneous than previously thought and that genetic and/or acquired factors may partially modulate the neurologic phenotype. Type 1 diabetes, and less frequently type 2, are considered early manifestations of ACP, occurring in 68.5% of patients at a median age of 38.5 years [109]. Similarly, anemia and/or microcytosis are common and early manifestations in ACP. In fact, they are reported in 80% of patients in Japan [102] and were the earliest symptoms in 86% of patients in Italy, decades earlier than diagnosis [108]. Although ACP is rare, Cp measurement should, therefore, be considered in patients with anemia, hyperferritinemia, and diabetes. This would enable early diagnosis and hopefully prevent development of neurological abnormalities, the most serious complication of the disease. At present, however, the treatment of ACP is still unsatisfactory. The main focus is on use of iron chelators (deferasirox, deferoxamine, and deferiprone) to reduce iron overload, which are effective in reducing systemic iron overload but have little effect on neurologic symptoms [73]. In addition, chelating agents may aggravate functional iron deficiency anemia and must often be misdrawn, which limits the long-term treatment needed to mobilize iron from the brain. Therefore, the period between the first symptoms and the onset of neurologic symptoms should be used to seek effective treatments to prevent or relieve neurologic symptoms [108,109]. Recently, intraperitoneal administration of CP to Cp-KO mice has been shown to cross the barrier and penetrate into the brain, restore ferroxidase activity, reduce iron accumulation in the brain, restore neuronal loss, and improve ataxia [110]. Fresh-frozen plasma administration as a source of CP was tentatively proposed in a couple of patients with partial and temporary restoring of iron metabolism, improvement in neurological symptoms, and brain iron deposition [111]. However, the short half-life of Cp (about 5 days) limits long-term therapy but leaves open the option of enzyme replacement therapy if ceruloplasmin becomes available for human use.

### 6.5. Gaucher Disease (OMIM #230800)

Gaucher disease (GD) is a rare, autosomal, recessive genetic disease caused by mutations in the *GBA1* gene. The activity of the lysosomal enzyme, glucocerebrosidase (GCase), is markedly decreased and the substrate glucosylceramide accumulates in macrophages, inducing their transformation in Gaucher cells. These lipid-containing macrophages are found primarily in the spleen, liver, and bone marrow and result in a complex disease with a heterogeneous clinical picture (reviewed in [112]). Gaucher disease is characterized by hepatosplenomegaly, cytopenia, sometimes severe bone involvement, and, in some forms, neurologic deficit. Diagnosis of GD was usually based on demonstrating insufficient GCase activity in whole leukocytes or monocytes, or in cultured fibroblasts. However, it has recently been proposed to change the diagnostic approach based on lyso-Gb1 measurements in dried blood spot (DBS) combined with *GBA1* genetic assays, without the need to confirm the diagnosis by measuring GCase enzyme activity [113]. More than 400 mutations have been described in *GBA1*, but some of them are more common, such as c.1226A > G (N370S), c.1448T > C (L444P), c.84dup, c.115 + 1G > A (IVS2 + 1G > A), and recombination events between GBA1 and its highly homogeneous pseudo gene (*GBA1P*) [114]. In general, three main phenotypic manifestations can be distinguished: Type-1 GD (GD-1), which is the most common form of the disease (90–95%) and is usually named non-neuronopathic GD; type-2 and type-3 are termed neuronopathic-GD. Several reports have documented hyperferritinemia in GD-1 patients whose serum ferritin levels may exceed 2–3 times the upper normal level [115,116]. More than 80% of adult patients with GD-1 have hyperferritinemia with normal serum iron and TSAT [117,118]. Although the pathophysiology of hyperferritinemia in GD-1 has not yet been elucidated, several studies point to the central role of macrophages. The broad spectrum of hyperferritinemia in GD-1 appears to be related to disease severity and is reversed by specific enzyme replacement therapy (ERT), suggesting a relationship between hyperferritinemia and lipid accumulation in Gaucher cells [117,118]. Lefebvre et al. [119], studying the interaction of iron proteins in in vitro models of Gaucher cells, found that alteration of local hepcidin–ferroportin interaction leading to down-regulation of ferroportin was implicated in iron sequestration, possibly explaining the mechanism of hyperferritinemia in GD-1 patients. Accordingly, iron accumulation is commonly observed in Gaucher cells, while only a small subset of GD-1 patients develop clinical iron overload [115,118]. Other authors have suggested that the chronic low-grade inflammation present in GD-1 may lead to dysregulation of ferroportin expression [116]. Studying macrophages derived from peripheral mononuclear cells from patients with type 1 Gaucher disease with genotype N370S/N370S, Aflaki et al. [120] found increased secretion of interleukins IL-1b and IL-6. They also demonstrated that impaired autophagy in Gaucher macrophages leads to inflammasome activation. They suggested that a link between lysosomal storage, impaired autophagy, and inflammation may contribute to clinical manifestations of GD-1 and hyperferritinemia through local cytokine activations or impairment of the nonclassical lysosomal secretory pathway of ferritin. In addition to the contribution of macrophages, some evidence suggests that defects within the erythroid compartment may also be involved in physiological dysfunctions of GD-1. Indeed, erythrocytes from patients exhibit altered biological phenotypes [121,122], and previous studies have shown that aberrant erythrocyte phagocytic events occur in bone marrow of patients with GD-1 [123,124]. Thus, it was recently shown that erythrocytes from patients with GD-1 are prone to phagocytosis by macrophages and that the consequent altered macrophage phenotype may contribute to development of Gaucher cells [113]. Phagocytosis of erythrocytes then facilitates iron retention by macrophages, increased ferritin synthesis, and secretion.

### 6.6. Unexplained Hyperferritinemia

Despite careful clinical and genetic investigations, the cause of isolated hyperferritinemia remained undetermined in some individuals. In 2017, Ravasi et al. described twelve Italian patients with unexplained isolated hyperferritinemia [125]. There were no mutations in *FTL* and *SLC40A1*, ruling out HHCS, benign hyperferritinemia, and ferroportin disease. The normality of L-ferritin concentrations in lymphomonocytes and red blood cells unlike those observed in patients with HHCS ruled out the presence of abnormalities in transcriptional and post-transcriptional ferritin regulation as a cause of hyperferritinemia. Unlike benign hyperferritinemia, the degree of glycosylation of serum ferritin was relatively low, although it was uncertain whether this finding had any significance in explaining hyperferritinemia. Although the cause of this form of hyperferritinemia has been difficult to determine, the case of four affected siblings suggests the presence of a recessive inherited disorder due to mutations in gene(s) not directly related to iron metabolism that might affect ferritin turnover, including ferritin secretion or clearance. Interestingly, a recent GWAS meta-analysis identified 46 new loci affecting iron homeostasis [126]. Many of the novel candidate genes play a role in iron homeostasis through mechanisms such as absorption, iron recycling, erythropoiesis, and hepcidin regulation, and some currently have no known function or have a function not directly related to iron metabolism [126]. Further studies are needed to elucidate the genetic cause(s) of unexplained forms of hyperferritinemia.

## 7. Conclusions

Inherited isolated hyperferritinemia includes several rare or ultra-rare disorders caused by mutations in genes directly involved in iron metabolism (*FTL*, *CP*, *SLC40A1*) or not, such as *GBA1*. They have autosomal dominant (HHCS, benign hyperferritinemia, ferroportin disease) or recessive phenotypes (aceruloplasminemia, Gaucher disease, and a yet unidentified form of hyperferritinemia). Diagnosis of these forms requires careful evaluation of patient and family history, patient clinical symptoms, and laboratory, genetic, and instrumental tests, which must be used according to a stepwise approach to distinguish them from the most common acquired causes of isolated hyperferritinemia. This approach is essential to avoid misdiagnosis, useless tests and therapies, and to provide patients with correct and adequate information about the diseases, their familial implication, follow-up, and possible therapies. In some patients, however, the cause of isolated hyperferritinemia remains unclear despite extensive investigation, suggesting the existence of genetic or acquired causes that have not yet been identified. Indeed, there are many unresolved questions regarding serum ferritin: a. the molecular pathways of ferritin secretion, clearance, and intracellular trafficking; b. the contribution of serum ferritin as an iron donor to the cell; c. the molecular mechanisms underlying the relationship between isolated hyperferritinemia and dysmetabolic changes and fatty liver. Studies aimed at clarifying these issues could lead to identification of new candidate proteins involved in ferritin metabolism and new possible markers and causes of isolated hyperferritinemia.

## Figures and Tables

**Table 1:** Causes and pathogenetic mechanisms of hyperferritinemia divided according to transferrin saturation.

**Transferrin saturation ≤ 45%**	
*Hyperferritinemia without iron overload*	*Pathogenetic mechanisms*
Inflammation and infection	Cytokine-dependent ferritin synthesis, hepcidin up-regulation, and macrophage iron retention
Hepatocellular necrosis	Ferritin leakage from damaged hepatocytes
Dysmetabolic Hyperferritinemia	Still unclear (sub-clinical inflammation; liver damage; hyperinsulinemia)
High alcohol intake	Alcohol-induced ferritin synthesis and ferritin leakage liver from damaged hepatocytes
Hyperthyroidism	Thyroid-hormone-mediated reduction in IRP binding to IRE
HHCS	Dysregulation of the IRE/IRP system
Benign Hyperferritinemia	Altered ferritin secretion or clearance
Gaucher disease	Iron sequestration in Gaucher’s cells
*Hyperferritinemia with iron overload* (*mostly mild or moderate*)	
Chronic liver diseases and PCT	Liver cell necrosis and inflammation; acquired and genetic cofactors favoring iron accumulation
Dysmetabolic Iron Overload Syndrome	Inflammation and liver damage
Transfusion-dependent (early stages)	Senescent RBC macrophage phagocytosis
Parenteral iron administration (inadequate)	Macrophage iron retention
Hemolytic anemias	Senescent RBC macrophage phagocytosis
Welding fume exposure (early stages)	Macrophage accumulation of iron nanoparticles
Ferroportin disease	Macrophage iron retention due to loss-of-function mutations of *SLC40A1*
Aceruloplasminemia	Reduced cellular iron export due to *CP* biallelic mutations
Gaucher disease type 1	Iron sequestration in Gaucher’s cells
**Transferrin saturation > 45%**	
*Primary Iron Overload*	*Pathogenesis*
Hemochromatosis (type 1, 2, 3)	Absent or reduced hepcidin synthesis due to biallelic mutations of *HFE*, *HAMP*, *HJV*, *TFR2*
Hemochromatosis type 4	Hepcidin resistance due to gain-of-function mutations of *SLC40A1*
PIGA disease	Reduced hepcidin and ceruloplasmin synthesis
*Secondary Iron Overload*	
Iron-loading anemias	Ineffective erythropoiesis inhibiting hepcidin synthesis
Chronic liver diseases (end stages)	Reduced synthesis of hepcidin and transferrin due to hepatic failure
Transfusion-dependent (late stages)	Massive iron overload from senescent RBC overwhelming macrophages capacity
Welding fume exposure (late stages)	Massive iron overload overwhelming macrophages capacity
*Other Iron Overload Diseases*	
African iron overload	Dietary habits, genetic susceptibility
Gestational alloimmune liver disease (GALD)	Liver damage and failure
Perinatal Hemochromatosis (other causes)	Liver damage and failure
Hypotransferrinemia	Increased NTBI-LPI accumulation due to biallelic mutations of *TF*
DMT1 deficiency	Derangement of cell iron trafficking in erythroblasts due to biallelic mutations of *DMT1*

*CP*: ceruloplasmin gene; DMT1: divalent transporter metal 1; HHCS: hereditary hyperferritinemia cataract syndrome; RBC: red blood cells; *PIGA*: phosphatidylinositol glycan anchor biosynthesis class A gene; NTBI: non-transferrin-bound iron; LPI: labile plasma iron.

**Table 2:** Epidemiology, genetics, most relevant clinical and biochemical characteristics, and therapeutical approach in inherited isolated hyperferritinemia.

**Hereditary Hyperferritinemia Cataract Syndrome**
Epidemiology: worldwide distribution; undefined prevalence (around 1:200,000); age of presentation 1–45 years
Genetics: dominant phenotype; single point mutation or deletions in the IRE region of FTL.
Clinical presentation: early cataract (generally before the age of 50 years);
Biochemical presentation: hyperferritinemia with normal TSAT
Therapy: no therapy for hyperferritinemia; cataract surgery when needed
**Benign Hyperferritinemia**
Epidemiology: described in Caucasians; undefined prevalence; age of presentation: adulthood
Genetics: dominant phenotype; three single point mutations identified (p.Gln26Leu, p.Ala27Val, p.Thr30Ile) in the first exon of FTL
Clinical presentation: no clinical manifestations reported
Biochemical presentation: hyperferritinemia with normal TSAT
Therapy: no therapy is needed
**Ferroportin disease**
Epidemiology: allele prevalence range 0.00034 to 0.0009; worldwide distribution; no gender prevalence; age of presentation: 20–60 years.
Genetics: dominant phenotype with incomplete penetrance and variable expression at both biochemical and clinical level; single point mutations, small deletions.
Clinical presentation: frequently asymptomatic; no clear conclusions regarding the pathogenic relationship between iron accumulation and liver damage.
Biochemical presentation: normal TSAT with variably increased serum ferritin. Increased TSAT may occur in later stages
Therapy: phlebotomies should be tailored according to liver iron accumulation in the induction phase (every 2–8 weeks), and every 4–12 months in the maintenance phase. Careful monitoring of Hb, TSAT, and ferritin to avoid development of iron-restricted erythropoiesis and anemia
**Aceruloplasminemia**
Epidemiology: estimated prevalence 1:2,000,000; worldwide distribution; no gender prevalence; age of presentation: 20–60 years.
Genetics: recessive phenotype with variable expression; single point mutations.
Clinical presentation: microcytic anemia, diabetes, retinal degeneration, wide spectrum of neuropsychiatric symptoms. Anemia and diabetes may precede neurological manifestations even by decades.
Biochemical presentation: mild microcytic anemia, low serum iron and TSAT, variably increased serum ferritin and hyperglycemia that may precede neurological manifestations by decades.
Therapy: iron chelators are effective in removing iron excess in the liver, but less or not at all effective in removing brain iron excess; dosage and frequency are matched to liver iron overload but are generally lower than in transfusion-dependent iron overload; deferiprone and deferasirox are preferred as they are better able to cross the blood–brain barrier. Check Hb and TSAT every month to prevent anemia, SF every 3–6 months. Plasma transfusion as a source of ceruloplasmin is under investigation.
**Gaucher disease type 1**
Epidemiology: prevalence between 1:40,000 and 1:60,000; worldwide distribution; age of onset: variable (the average onset of disease of patients in the Gaucher registry is 20.4 years)
Genetics: recessive phenotype with variable penetrance and expression; more than 400 mutations described, including single point mutations, small deletion, and extensive rearrangements.
Clinical presentation: fatigue, growth retardation, and delayed puberty in children, splenomegaly (>90%) and hepatomegaly (60–80%), bleeding manifestations, painful bone crisis (more common in children), increased risk of developing Parkinson’s disease
Biochemical presentation: thrombocytopenia (90%), moderate anemia (20–40%), polyclonal hypergammaglobulinemia, and monoclonal gammopathy.
Therapy: enzyme replacement therapy (ERT), substrate reduction therapy (SRT)
**Unexplained isolated hyperferritinemia**
Epidemiology: undefined
Genetics: autosomal recessive; gene still to be identified
Clinical presentation: no clinical manifestations reported
Biochemical presentation: unexplained hyperferritinemia with normal TSAT without iron overload
Therapy: no therapy is needed

## Data Availability

Not applicable.

## Abbreviations

BK	bradykinin
GD-1	Gaucher Disease
GWAS	Genome-Wide Association Studies
HEIRS	Hemochromatosis and Iron Overload Screening
HHCS	Hereditary Hyperferritinemia Cataract Syndrome
HK	High-molecular-weight kininogen
HKa	two-chain high-molecular-weight kininogen
IRE	Iron-Responsive Element
IRP	Iron Regulatory Protein
IL-1	interleukin-1-β
LPS	Lipopolysaccharide
LROs	secretory lysosomes or secretory autophagy
LIP	Labile Iron Pool
NTBI	Non-Transferrin-Bound Iron
TNF-α	tumor necrosis factor-α
TSAT	Transferrin SATuration
